# Psychological outcomes and associated factors amongst healthcare workers during a single wave, deeper into the COVID-19 pandemic in China

**DOI:** 10.3389/fpsyt.2022.983909

**Published:** 2022-10-06

**Authors:** Jianyong Tang, You Wu, Hongyan Qi, Dongjing Li, Jianfei Shi, Wei Wang, Mengmeng Niu, Liang Liu, Dong Wang, Xia Li

**Affiliations:** ^1^Department of Laboratory Medicine, Xi'an Children's Hospital, The Affiliated Children's Hospital of Xi'an Jiaotong University, Xi'an, China; ^2^Department of Laboratory Medicine, National Children's Medical Center for Northwest Region, Xi'an, China; ^3^Department of Neurology, Xi'an Children's Hospital, The Affiliated Children's Hospital of Xi'an Jiaotong University, Xi'an, China; ^4^Department of Neurology, National Children's Medical Center for Northwest Region, Xi'an, China

**Keywords:** mental health, healthcare workers, COVID-19, China, single wave

## Abstract

**Background:**

To date, the repeated breakout of the novel coronavirus disease 2019 (COVID-19) pandemic across many regions in China has caused continuous physical and mental harm to health care workers. This study investigates the psychological burden of the pandemic and its associated risk factors among Chinese healthcare workers (HCWs) during a single wave of COVID-19.

**Methods:**

For this cross-sectional web-based survey conducted from January 16, 2022 to February 5, 2022, a total of 412 HCWs from Northwestern China were recruited. Their socio-demographic data and COVID-19 related survey variables were then collected using online self-rating questionnaires. In addition, the Chinese versions of well-validated instruments, including the 12-item General Health Questionnaire for psychiatric morbidity, the Generalized Anxiety Disorder Scale-7 for anxiety, the Patient Health Questionnaire-9 for depression and the Insomnia Severity Index-7 for insomnia, were used to assess the participants' mental health status. Multivariate logistic regression analysis was eventually performed to identify the risk factors associated with the psychological outcomes.

**Results:**

Of the 388 participants who were included in the final study (94.17% response rate), the prevalence of anxiety, depression, and insomnia symptoms were 25.3% (95% CI: 20.9-29.6%), 40.7% (95% CI: 35.8-45.6%), and 30.9% (95% CI: 26.3-35.5%), respectively. Multivariate logistic regression analysis revealed that being a woman and having a perceived need for psychological support were risk factors for all psychological outcomes, while poor disease cognition and perceived susceptibility were risk factors for anxiety. Poor disease cognition and being unvaccinated against COVID-19 were risk factors for depression, with the latter also being an independent risk factor for insomnia.

**Conclusion:**

This study has identified a relatively lower prevalence rate of psychological disorders among Chinese HCWs during a single wave, deeper into the COVID-19 pandemic. Female HCWs, and those who had a perceived need for psychological support, had poor disease cognition, were perceived as susceptible to COVID-19 and had not been vaccinated against COVID-19 deserve more attention.

## Introduction

At the end of December 2019, the city of Wuhan of Hubei Province, China reported the first cases of the novel coronavirus disease 2019 (COVID-19) (1). The disease then rapidly spread to countries around the world, causing the World Health Organization to declare it as a pandemic on 11 March 2020 (2). Although COVID-19 vaccination has gradually become popular and effective preventive measures have become increasingly standardized, the constant variability and highly infectious nature of the severe acute respiratory syndrome coronavirus 2 (SARS-CoV-2) responsible for the disease cause the number of COVID-19 infections and deaths to rise continuously worldwide. In fact, as of February 20, 2022, over 422 million confirmed cases and over 5.8 million deaths had been reported around the world (3). In China, since the initial outbreak, repeated waves of the pandemic have occurred in many cities (e.g., Nanjing, Guangzhou, Lanzhou, Zhengzhou, Xi'an, Hongkong), with a total of 174 486 confirmed cases and 5,776 deaths reported until February 20, 2022 (3). These repeated outbreaks pose a serious challenge to the health services by constantly imposing physical and mental pressure on healthcare workers (HCWs).

During the COVID-19 pandemic, a large number of HCWs had volunteered to assist in fighting the impact of the pandemic. However, at the same time, HCWs had become more prone to mental health problems due to a number of factors including but not limited to work overload, high-pressure working environments, insufficient personal protective equipment, scarcity of effective therapeutic regimen, excessive media coverage and feelings of inadequate support (4, 5). In fact, several studies in China have confirmed that the medical personnel experienced mental health symptoms (e.g., anxiety, insomnia, depression, distress) during the early period of the pandemic (6–8). Thus, greater attention needs to be given to the mental health of HCWs. In 2020, the National Health Commission issued a public document requiring that all localities should focus on the mental health of the medical personnel and strengthen psychological assistance services (9). However, little research has been done on the psychological well-being of healthcare workers during the waves which occurred deeper into the COVID-19 pandemic in China. Hence, the present study sought to investigate the mental health burden among Chinese HCWs during a single wave, deeper into the pandemic while identifying the risk factors associated with these mental health issues. It is expected that this will help to provide evidence that will guide potential psychological interventions for healthcare workers.

## Materials and methods

### Study design and participants

This cross-sectional study was conducted online *via* the “questionnaire star” platform from January 16, 2022 to February 5, 2022 using a convenience sampling method for participant selection. The online survey link was sent to prospective participants, consisting of healthcare workers (physicians, medical technicians, nurses and administrator/rear-service staffs) from different hospitals across Northwestern China, through WeChat and only one set of responses could be generated from one WeChat account. The exclusion criteria for invalid questionnaires were as follows: (a). more than 2/3 of the total questions were missed/unanswered; (b). the same item was selected for all questions; (c). items selected in the questionnaire followed a fixed pattern (e.g., select 1,2,1,2,1,2,1,2, … for all questions); (d). there were irrational answers in the questionnaire (e.g., 20–30 years old for age and >20 years for work experience). All participants were fully aware of the study's purpose prior to the start of the survey, with participation indicating consent. Prior ethics approval was sought from the Medical Ethics Committee of the Affiliated Children's Hospital of Xi'an Jiaotong University (Approval Number: 20220046) before planning and conducting the study as required by the Declaration of Helsinki.

### Measurements

All participants were required to answer a socio-demographic questionnaire to provide details on gender, age, relationship status, profession, title of technician, highest academic degree obtained, work experience, if they had children and underlying diseases. In addition, answers to the following six COVID-19-related questions were required: (1). Getting vaccinated: Are you vaccinated against COVID-19? (2). Disease cognition: Do you know about COVID-19 and its treatment? (3). Fear of infection: Are you afraid of contracting COVID-19? (4). Contacting history: Have you ever been in contact with confirmed or suspected cases of COVID-19? (5). Perceived susceptibility: Do you think that you are vulnerable to the novel coronavirus? (6). Psychological support: Do you have a perceived need for psychological support? Each question was rated on a dichotomous scale (“yes” or “no”).

This study also used Chinese versions of the following well-validated instruments to assess the psychological morbidity (e.g., the symptoms of anxiety, depression, or insomnia) of all participants: the 7-item Insomnia Severity Index (ISI-7), the 9-item Patient Health Questionnaire (PHQ-9), the 7-item Generalized Anxiety Disorder Scale (GAD-7) and the 12-item General Health Questionnaire (GHQ-12) (10–13).

As a self-rating questionnaire which uses a 4-point Likert scale (from “never” to “always”) for each item, the GHQ-12 is largely used to identify general mental issues (14). Based on previous studies, a bimodal scoring method (0-0-1-1) was used for participants' responses. Thus, for the 12 items, the GHQ-12 total score could range from 0 to 12, with scores of 4 and above indicating a likelihood of psychiatric morbidity (15).

The other scales, namely ISI-7, PHQ-9 and GAD-7 were applied for respectively measuring the level of insomnia, depression and anxiety among the participants. For both the GAD-7 and PHQ-9, a 4-point scale was used for each item, with the options ranging from “0” (not at all) to “3” (nearly every day). This provided total scores of 0–21 for GAD-7 (seven items) while for the latter, the scores ranged from 0 to 27. From these scores, the severity of anxiety symptoms could subsequently be classified into the following four levels, with the score range corresponding to those levels shown in parentheses: absence of anxiety (0–4), mild anxiety (5–9), moderate anxiety (10–14) and severe anxiety (15–21) (11). Similarly, for depression symptoms, overall scores could be classified into the following five severity levels: absence of depression (0–4), mild depression (5–9), moderate depression (10–14), moderately severe depression (15–19) and severe depression (20–27) (12). As far as the ISI-7 scale was concerned, it was a simple measurement tool that screened for insomnia by assessing the nature and severity of sleep disturbance based on seven items. Responses for each item were rated on a 5-point Likert scale (0 = “none”, 4 = “very”) which yielded total scores between 0 and 28 and in this case, the severity of insomnia could be classified as follows: absence of insomnia (0–7), sub-threshold insomnia (8–14), moderate insomnia (15–21) and severe insomnia (22–28) (13). Moreover, the cut-off scores for identifying psychiatric morbidity, anxiety, depression and insomnia disorder were 4, 7 (16), 10 and 15, respectively.

### Statistical analysis

IBM SPSS Statistics for Windows, version 25.0 (IBM Corp, Armonk, NY), was used to analyze all data, with socio-demographic as well as COVID-19-related data summarized and presented as frequencies (percentage), means and standard deviations or medians and interquartile ranges depending on the data distribution. In addition, scores from the GHQ-12, GAD-7, PHQ-9 and ISI-7 scales for two or more groups were compared using independent-samples *t*-tests as well as one-way analysis of variance. These were then followed by an LSD *t*-test or the nonparametric Mann–Whitney *U*-test and Kruskal-Wallis H-test, with results considered to be statistically significant for *p*-values < 0.05. The tolerance and variance inflation factor (VIF) tests were also applied to detect the multicollinearity of variables and no multicollinearity was found. Finally, potential risk factors associated with psychological outcomes were identified through multivariate analyses with binary logistic regression models (enter model, forward elimination method, and backward elimination method) (15). All independent variables were included in the models for binary logistic regression analysis firstly, and the factors which converged well were included in the initial models while the estimates did not converge were excluded. Then, the significant factors (which converged well in all three initial models and significant in at least one model) identified from the initial logistic models were included in the final models, and the final independent risk factors were identified. In this case, possible links between risk factors and outcomes were expressed as odds ratios (ORs) and 95% confidence limits (CIs).

## Results

### Socio-demographics characteristics and COVID-19 related surveys

Of the 412 HCWs who took part in the study, questionnaires from 24 were considered to be invalid and were therefore excluded. For the remaining 388 participants (representing 94.17% of the responses) included in the final sample, 177 (45.6%) were males, 211 (54.4%) were females and a large proportion of the individuals were married [321 (82.7%)]. The participants' age varied between 20 and 59 years old, with most being between 31 and 40 years old [22 (57.0%)]. Regarding their occupation, more than half of the respondents were physicians [222 (57.2%)] while the number of medical technicians, nurses and administrator/rear-service staffs were 66 (17.0%), 74 (19.1%), and 26 (6.7%), respectively. Furthermore, most of the participants also had a medium-grade technical title [184 (47.4%)], a bachelor's degree as the highest education level [206 (53.1%)] and a work experience of < 10 years [192 (49.5%)]. Finally, nearly three-quarter of the participants [287 (74.0%)] had children, and less than one-tenth of the sampled individuals [34 (8.8%)] had underlying diseases (Table 1).

**Table 1:** Socio-demographic characteristics and COVID-19 related surveys of respondents.

**Characteristics**	**Category**	***n* (%)**
Gender	Male	177 (45.6)
	Female	211 (54.4)
Age (year)	20~30	106 (27.3)
	31~40	221 (57.0)
	41~50	49 (12.6)
	>50	12 (3.1)
Relationship status	Married	321 (82.7)
	Single	67 (17.3)
Occupation	Physician	222 (57.2)
	Medical technician	66 (17.0)
	Nurse	74 (19.1)
	Administrator/Rear-service	26 (6.7)
Title of technician	Senior level	78 (20.1)
	Medium level	184 (47.4)
	Junior level	126 (32.5)
Highest academic degree obtained	Doctor's degree	13 (3.4)
	Master's degree	141 (36.3)
	Bachelor's degree	206 (53.1)
	Associate degree or below	28 (7.2)
Work Experience (year)	< 10	192 (49.5)
	10~20	155 (39.9)
	>20	41 (10.6)
Having children	YES	287 (74.0)
	NO	101 (26.0)
Underlying diseases	YES	34 (8.8)
	NO	354 (91.2)
Getting vaccinated	YES	357 (92.0)
	NO	31 (8.0)
Disease cognition	YES	340 (87.6)
	NO	48 (12.4)
Fear of infection	YES	248 (63.9)
	NO	140 (36.1)
Contacting history	YES	58 (14.9)
	NO	330 (85.1)
Perceived susceptibility	YES	120 (30.9)
	NO	268 (69.1)
Psychological support	YES	60 (15.5)
	NO	328 (84.5)

In terms of responses to COVID-19-related questions, of the 388 participants included, 357 (92.0%) had been vaccinated, 340 (87.6%) had full disease cognition and 248 (63.9%) had a fear of infection. However, only 58 (14.9%) of the respondents had been in contact with confirmed or suspected cases of COVID-19, while the number of individuals who were perceived as susceptible to COVID-19 and who had a perceived need for psychological support were 120 (30.9%) and 60 (15.5%) respectively (Table 1).

### Severity levels of anxiety, depression, and insomnia

**Figure 1:**
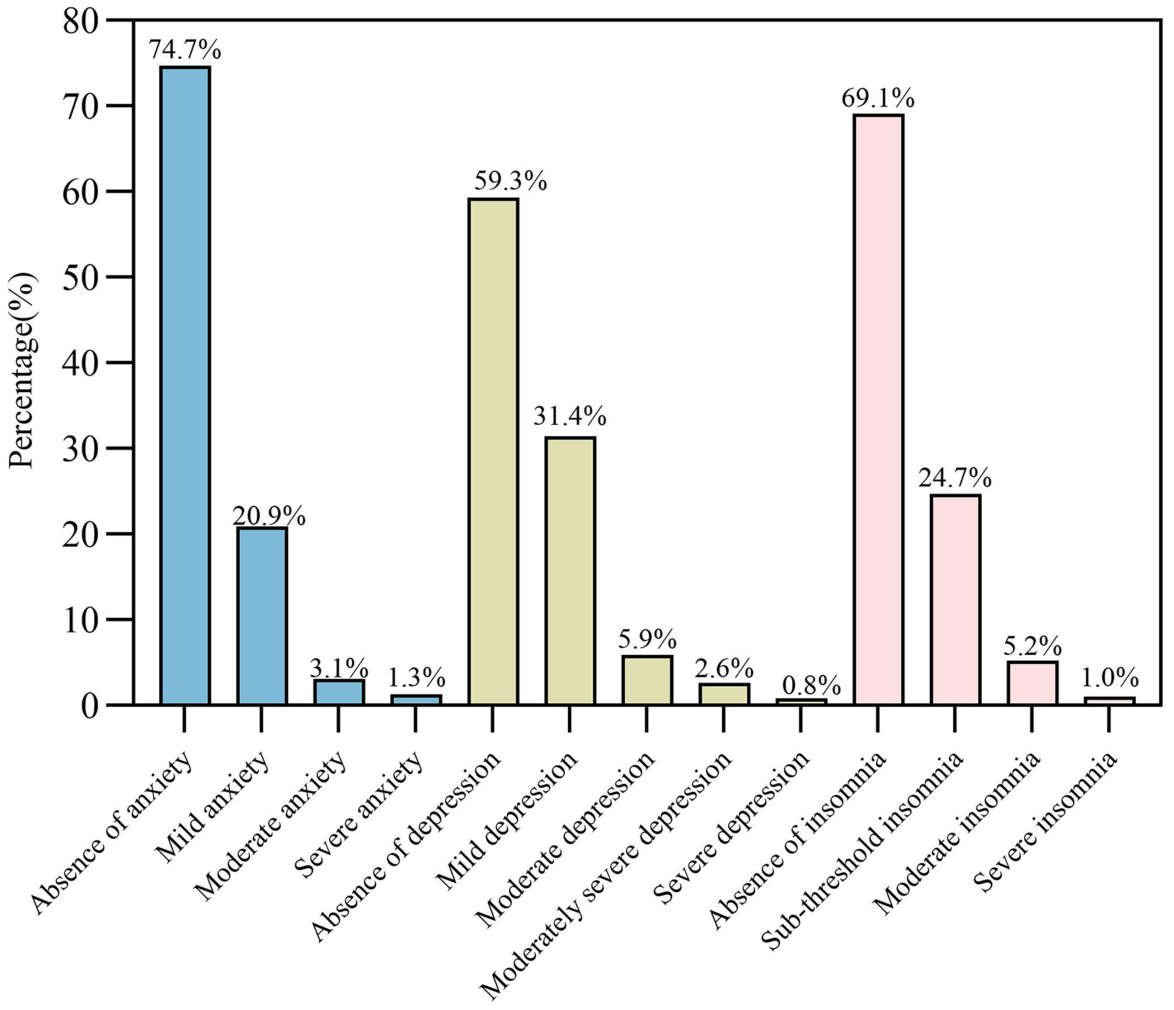
Severity levels of anxiety, depression and insomnia among HCWs.

Based on the total scores from the GAD-7, PHQ-9 and ISI-7 scales, 98 (25.3%, 95% CI: 20.9–29.6%) of the participants had anxiety symptoms, 158 (40.7%, 95% CI: 35.8–45.6%) were identified as having depressive symptoms and 120 (30.9%, 95% CI: 26.3–35.5%) were found to have insomnia. In terms of anxiety levels, 81 (20.9%) experienced mild anxiety, 12 (3.1%) experienced moderate levels of anxiety and only 5 (1.3%) experienced severe ones. In addition, the scores for the PHQ-9 scale showed that 122 (31.4%), 23 (5.9%), 10 (2.6%) and 3 (0.8%) participants experienced symptoms of mild, moderate, moderately severe and severe depression respectively while in the case of insomnia, symptoms of mild, moderate and severe insomnia were identified for 96 (24.7%), 20 (5.2%) and 4 (1.0%) participants, respectively (Figure 1).

### Univariate analysis

For the univariate analysis, it was observed that the GHQ-12 scores for females were significantly higher compared to those of males (*t* = −3.990, *p* < 0.001), with differences at gender level also found regarding the scores for ISI-7 (*t* = −3.999, *p* < 0.001), PHQ-9 (*t* = −4.022, *p* < 0.001) and GAD-7 (*t* = −2.873, *p* = 0.004). Furthermore, symptoms of depression varied significantly between professional titles (*F* = 3.644, *p* = 0.027) and work experience (*F* = 3.205, *p* = 0.042). Participants with medium level of technician title and < 10 years of work experience had the highest scores on PHQ-9, while those with senior titles and more than 20 years of work experience had the lowest scores. Meanwhile, higher scores were obtained on the PHQ-9 (*t* = 4.055, *p* < 0.001) and ISI-7 (*t* = 2.952, *p* = 0.003) scales by those who had children. Finally, participants with underlying diseases scored significantly higher for the ISI-7 (*t* = 2.880, *p* = 0.004), PHQ-9 (*t* = 2.952, *p* = 0.003) and GAD-7 (*t* = 2.043, *p* = 0.042) scales compared with those without diseases (Table 2).

**Table 2:** Association of GHQ-12, GAD-7, PHQ-9 and ISI-7 scores with demographic characteristics (*n* = 388).

**Characteristics**	**GHQ-12 score**,	**GAD-7 score**,	**PHQ-9 score**,	**ISI-7 score**,
	**mean (SD)**	**mean (SD)**	**mean (SD)**	**mean (SD)**
**Gender**	***	**	***	***
Male	1.03 (1.85)	2.40 (3.04)	3.32 (3.80)	4.63 (4.28)
Female	1.92 (2.53)	3.36 (3.50)	5.02 (4.51)	6.61 (5.44)
**Age (year)**	NS	NS	NS	NS
20~30	1.25 (1.88)	3.09 (3.25)	4.41 (4.15)	5.88 (5.09)
31~40	1.78 (2.53)	2.95 (3.49)	4.45 (4.42)	5.93 (5.14)
41~50	1.06 (1.97)	2.45 (2.64)	3.61 (4.09)	4.69 (4.54)
>50	0.83 (1.59)	2.75 (3.75)	1.58 (2.39)	4.17 (4.17)
**Relationship status**	NS	NS	NS	NS
Married	1.55 (2.33)	2.86 (3.37)	4.18 (4.28)	5.49 (4.92)
Single	1.36 (2.09)	3.19 (3.14)	4.55 (4.30)	6.76 (5.45)
**Occupation**	NS	NS	NS	NS
Physician	1.72 (2.48)	2.98 (3.12)	4.43 (4.18)	5.50 (4.86)
Medical technician	1.44 (2.37)	2.98 (4.17)	4.59 (4.92)	6.44 (5.20)
Nurse	1.27 (1.77)	2.68 (3.34)	3.62 (4.09)	6.08 (5.58)
Administrator/Rear-service	0.69 (1.38)	2.96 (2.74)	3.54 (3.92)	4.50 (4.26)
**Title of technician**	NS	NS	*	NS
Senior level	1.14 (1.72)	2.60 (2.79)	3.09 (3.30)	4.81 (4.27)
Medium level	1.76 (2.65)	2.90 (3.57)	4.59 (4.51)	5.65 (5.23)
Junior level	1.40 (1.97)	3.15 (3.28)	4.45 (4.37)	6.35 (5.13)
**Highest academic degree obtained**	NS	NS	NS	NS
Doctor's degree	1.46 (1.98)	2.62 (3.40)	4.38 (4.01)	6.23 (5.40)
Master's degree	1.82 (2.53)	3.23 (3.06)	4.74 (4.26)	5.65 (5.02)
Bachelor's degree	1.37 (2.20)	2.83 (3.59)	4.03 (4.26)	5.78 (5.11)
Associate degree or below	1.11 (1.60)	2.14 (2.48)	3.25 (4.54)	5.21 (4.51)
**Work experience (year)**	*	NS	*	NS
< 10	1.44 (2.06)	2.95 (2.99)	4.47 (4.18)	5.73 (4.88)
10~20	1.83 (2.69)	3.03 (3.80)	4.38 (4.50)	5.79 (5.36)
>20	0.73 (1.25)	2.34 (2.93)	2.66 (3.62)	5.27 (4.58)
**Having children**	***	NS	***	**
YES	1.71 (2.48)	3.09 (3.48)	4.66 (4.62)	6.08 (5.25)
NO	0.96 (1.50)	2.43 (2.80)	3.07 (2.83)	4.64 (4.20)
**Underlying diseases**	NS	*	**	**
YES	2.26 (3.05)	4.03 (3.90)	6.29 (5.75)	8.06 (6.78)
NO	1.45 (2.19)	2.81 (3.26)	4.05 (4.07)	5.48 (4.79)

^*^p < 0.05, ^**^p < 0.01, ^***^p < 0.001.

**Figure 2:**
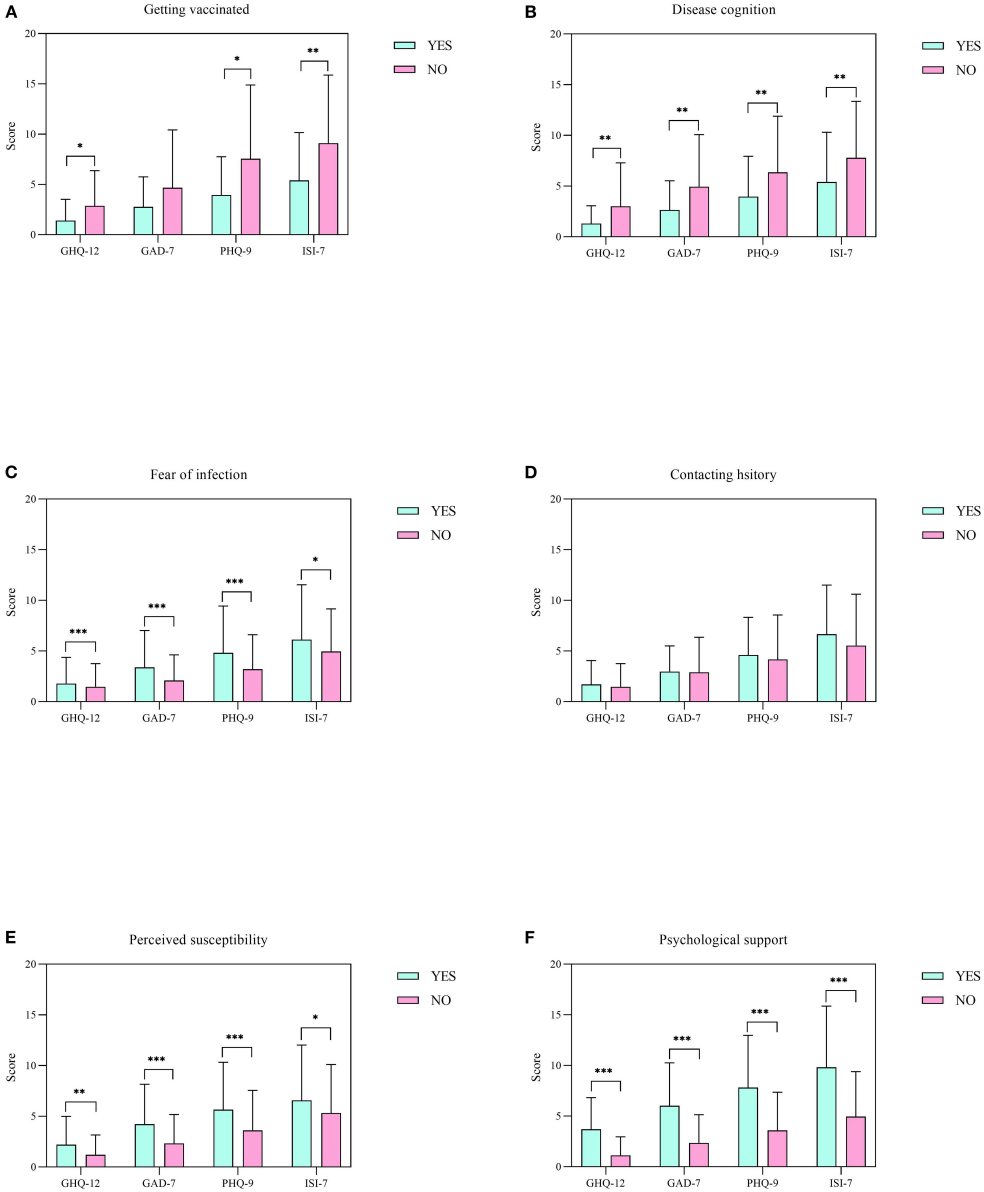
Comparison of the responses of HCWs for COVID-19-related surveys. **(A–F)** Scores on the GHQ-12, GAD-7, PHQ-9 and ISI-7 scales for getting vaccinated, disease cognition, fear of infection, contacting history, perceived susceptibility and psychological support among healthcare workers, respectively. All data represent means ± standard deviations (SD). *p < 0.05, **p < 0.01, and ***p < 0.001.

Regarding the COVID-19-related survey, significantly lower scores for GHQ-12, PHQ-9 and ISI-7 were obtained for respondents who had been vaccinated (*p* = 0.028, *p* = 0.011 and *p* = 0.006, respectively) and had full disease cognition (*p* = 0.009, *p* = 0.005 and *p* = 0.002, respectively), while significantly higher ones were noted for those who had a fear of infection (*p* < 0.001, *p* < 0.001 and *p* = 0.019, respectively), who were perceived as susceptible to COVID-19 (*p* = 0.001, *p* < 0.001 and *p* = 0.024, respectively) and who had a need for psychological support (*p* < 0.001, *p* < 0.001 and *p* < 0.001, respectively). However, concerning the GAD-7 scale, participants who had full disease cognition obtained significantly lower scores (*p* = 0.004), while significantly higher ones were obtained for those who had a fear of infection, were perceived as susceptible to COVID-19 and who had a perceived need for psychological support (*p* < 0.001, *p* < 0.001 and *p* < 0.001, respectively) (Figure 2).

### Multivariate logistic regression analysis

**Figure 3:**
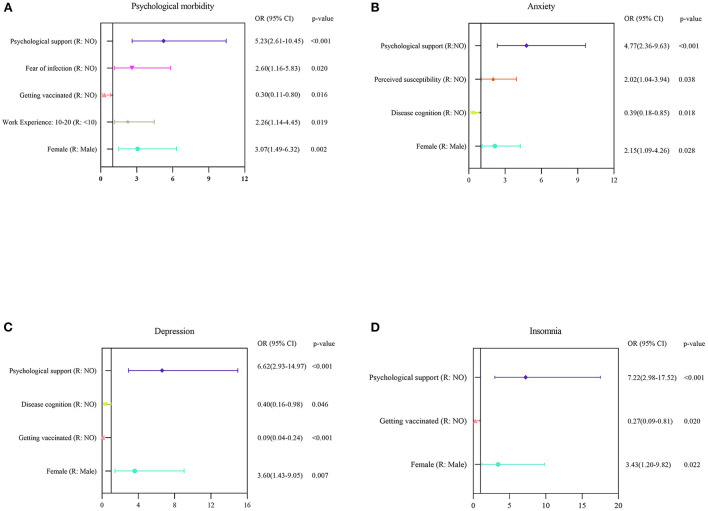
Multivariate logistic regression analyses for identifying the risk factors linked to mental health outcomes among HCWs. **(A–D)** Risk factors associated with general psychological morbidity, anxiety, depression, and insomnia, which identified based on the scores of GHQ-12, GAD-7, PHQ-9, and ISI-7, respectively.

Based on the previously-defined cut-off values, factors which were independently associated with psychiatric morbidity, anxiety, depression and insomnia, as determined by multivariable logistic regression analyses, are shown in Figure 3. The results revealed that being a woman and having a perceived need for psychological support were associated with all the mental outcomes under study (e.g., anxiety among women: OR: 2.15, 95% CI: 1.09–4.26, *p* = 0.028; depression among those with a perceived need for psychological support: OR: 6.62, 95% CI: 2.93–14.97, *p* < 0.001). Participants with 10~20 years of work experience were also more likely to develop psychiatric morbidity compared with those whose work experience was < 10 years (OR: 2.26, 95% CI: 1.14–4.45, *p*=0.019). Similarly, getting vaccinated was linked to lower risks of developing psychiatric morbidity, depression and insomnia (OR: 0.30; 95% CI: 0.11–0.80, *p* = 0.016; OR: 0.09; 95% CI: 0.04–0.24, *p* < 0.001; and OR: 0.27; 95% CI: 0.09–0.81, *p* = 0.020, respectively), while full disease cognition was linked to lower risks of feeling anxious and depressed (OR: 0.39; 95% CI: 0.18–0.85, *p* = 0.018; and OR: 0.40; 95% CI: 0.16–0.98, *p* = 0.046, respectively). Participants were also more likely to develop psychiatric morbidity due to fear of infection (OR: 2.60; 95% CI: 1.16–5.83, *p* = 0.020), while those having a perceived susceptibility to COVID-19 were at a higher risk of developing anxiety symptoms (OR: 2.02; 95% CI: 1.04–3.94, *p* = 0.038).

## Discussion

In this cross-sectional study, most of the participants had been vaccinated against COVID-19 (92.0%), had full disease cognition (87.6%) and had a fear of infection (63.9%). In addition, only a few had been in contact with suspected or confirmed cases of COVID-19 (14.9%), were perceived as susceptible to COVID-19 (30.9) and had a perceived need for psychological support (15.5%). Finally, according to the GHQ-12 scores, only 53 (13.7%) out of the 388 HCWs who completed the survey, reported general psychological issues.

The prevalence of anxiety, depression and insomnia reported for HCWs in the present study (25.3, 40.7, and 30.9%, respectively) were relatively lower compared with those identified by Lai et al. (44.6, 50.4, and 34.0%, respectively)(8) and by Zhang et al. (44.7, 50.7, and 36.1%, respectively) (17) in previous studies conducted between January and February 2020 in China. However, the prevalence of mental health outcomes, as obtained in this study, was similar to that reported by Tian et al. during a study done in April 2020 (20.7, 45.6, and 27.0%, respectively) (18). Furthermore, for studies involving HCWs in other countries and regions around the world, the prevalence of anxiety reported by Temsah et al. was 38.9% (19), while that of depression, reported by Yadeta et al. was 66.4% (20). Similarly, *Rossi* et al. found that 8.27% of the participants in their study had severe insomnia (21), with this value being higher than the 1.0% obtained in this study. These differences could have been due to the fact that most of these studies were carried out when the COVID-19 outbreak was still in its early stages. However, as the pandemic lasted, the large number of studies undertaken to identify the characteristics of the pathogen and the disease enabled HCWs to learn how to effectively protect themselves against the disease. Additionally, as prevention measures to mitigate the impact of the pandemic improved, the psychological pressure on HCWs was reduced. However, since COVID-19 was highly transmissible and was linked to high morbidity as well as potential death, it still represented a sustained psychological burden on HCWs (22).

Overall, the results suggested that psychological problems were more prevalent in female HCWs compared with males and this was in line with previous studies conducted in China (8), Ethiopia (20), Italy (21), India (19) and Egypt (23). Studies have shown that females tend to perceive themselves as more likely to fall into stressful circumstances than males (24). During a pandemic, they are also often among the vulnerable population due to differences in gender and social roles. Therefore, it is likely that female HCWs might require more attention and for this purpose, more gender-specific psychological intervention strategies need to be taken to address mental health inequities (25). Additionally, it was found that depression and insomnia were significantly linked to having children and underlying diseases, with similar findings reported by Maha et al. who pointed out that residing with families could predict increased depression and anxiety (26). Furthermore, underlying diseases were also significantly associated with anxiety symptoms. Indeed, having such diseases may cause HCWs to feel less confident and more vulnerable to COVID-19, thereby increasing their psychological distress. However, unlike previous studies, the current one did not find significant differences to be linked to age, relationship status, occupation and technician title of participants. Larger sample sizes could be applied in future studies to further support the absence of such links.

The current study also investigated how several COVID-19-related factors affected the psychological conditions of HCWs. It was observed that 15.5% of the respondents reported a perceived need for psychological support, with this factor significantly linked to all mental health outcomes among HCWs. Previous studies carried out on the 2003 SARS pandemic have shown that disease outbreaks can create long-term negative psychological impacts such as anxiety, depression, stress and even post-traumatic stress disorder on healthcare workers (27). Since the COVID-19 pandemic has lasted for more than 2 years since the first outbreak, continuous stimulation from recurring outbreaks could have caused serious long-lasting psychological harm to HCWs. However, unlike most previous studies, no significant association between a history of contacting COVID-19 and psychological problems was found in the current study. In fact, since the pandemic began, most HCWs have experienced notable challenges in their daily work, such as an upheaval in work patterns, a high-pressure working environment, increased workload and shift timings. This study demonstrated that the risk of adverse psychological reactions was not restricted to frontline HCWs because as the pandemic continues, the mental health of non-frontline HCWs also deserves attention.

The current results further revealed a significant link between the measured mental health outcomes and disease cognition or fear of infection. At the same time, only disease cognition was an independent risk factor for anxiety and depression symptoms. Poor disease cognition for COVID-19 may cause more extreme psychological responses (e.g., fear, worry, helpless) among HCWs, with subsequent overreactions further leading to poor mental health. This highlights the importance of more in-depth and comprehensive education regarding COVID-19 as well as the need to publicly provide COVID-19- related knowledge to HCWs in order to address their uncertainty and fears (28). A significant relationship was also noted between perceived susceptibility and anxiety, depression and insomnia, but perceived susceptibility was a significant predictor for anxiety only. These findings support those from previous studies performed during the early period of the COVID-19 pandemic (20, 29). Although the greater availability of personal protective equipment can alleviate feelings of fear, the long-lasting pandemic has been the source of burnout, causing HCWs to show psychological slackness in their personal protection. This, in turn, could have increased perceptions of risks and susceptibility (30).

Finally, vaccination against COVID-19 was found to be a significant protective factor against the symptoms of anxiety and insomnia, indicating that HCWs could benefit from vaccination to reduce risks of psychological problems. In addition to their safety and effectiveness, vaccines represent an economically viable means for preventing, controlling and even eliminating infectious diseases. Recognizing the importance of vaccination and increasing the vaccination rate in society could be helpful to end the pandemic as vaccines are not only effective in preventing the spread of infections but also in addressing pandemic-related fears and subsequent mental health problems among HCWs, as well as the general population (31).

## Strengths and limitations

The main strength of the current study was that it applied validated measurement tools with good reliability to assess the psychological problems which healthcare workers faced during a single wave that occurred deeper into COVID-19 pandemic in China, with these tools helping to identify the risk factors associated with these psychological issues. Most of the previous studies on mental health outcomes among HCWs were carried out during the early period of the pandemic, while the current one was concerned with similar outcomes which were encountered later into pandemic as this aspect was somewhat understudied. However, this study also contained some limitations and weaknesses. Firstly, with the convenience sampling method used being not very strict, this could have led to biased results. Secondly, most of the respondents in this study were from Northwestern China and as such, the sample was not representative of the population. Thirdly, the number of participants was limited, thereby making it difficult to generalize the results for the whole population of HCWs. Hence, larger samples of HCWs enrolled in multicenter studies would need to be considered to further verify the results. Fourthly, the online survey was also conducted without physician-led psychiatric evaluation, and as a result, it was not possible to determine if respondents had mental illnesses prior to the survey. Lastly, this study was a cross-sectional survey that could not accurately reflect the dynamic changes of mental health state. Thus, further investigations would be required to explore the pandemic's long-term impact on mental health among HCWs.

## Conclusion

This study revealed a relatively lower prevalence rate of psychological disorders amongst healthcare workers during a single wave deeper into the COVID-19 pandemic in China, thus indicating a higher psychological endurance to the pandemic compared with the early period. In general, being a woman and having a perceived need for psychological support were independent risk factors for all the mental health outcomes under study. At the same time, poor disease cognition and being perceived as susceptible to COVID-19 increased risks of anxiety. Similarly, participants who were unvaccinated against COVID-19 and who had poor disease cognition had a higher risk of depression, with being unvaccinated against COVID-19 also increasing the likelihood of insomnia. Overall, the findings highlight the need to develop more targeted intervention measures that seek to address the psychological problems of vulnerable HCWs.

## Data availability statement

The raw data supporting the conclusions of this article will be made available by the authors, without undue reservation.

## Ethics statement

The studies involving human participants were reviewed and approved by the Medical Ethics Committee of the Affiliated Children's Hospital of Xi'an Jiaotong University. The patients/participants provided their written informed consent to participate in this study.

## Author contributions

JT, YW, XL, and DW were involved in the conceptualization and design of this study. YW, HQ, DL, LL, and MN were involved in the data collection. JT, JS, and WW performed the statistical analyses. JT and YW wrote the first draft of the manuscript and all authors commented on previous versions of the article. All authors approved the final manuscript.

## Conflict of interest

The authors declare that the research was conducted in the absence of any commercial or financial relationships that could be construed as a potential conflict of interest.

## Publisher's note

All claims expressed in this article are solely those of the authors and do not necessarily represent those of their affiliated organizations, or those of the publisher, the editors and the reviewers. Any product that may be evaluated in this article, or claim that may be made by its manufacturer, is not guaranteed or endorsed by the publisher.
